# Activation of PI3K signaling prevents aminoglycoside-induced hair cell death in the murine cochlea

**DOI:** 10.1242/bio.016758

**Published:** 2016-05-03

**Authors:** Azadeh Jadali, Kelvin Y. Kwan

**Affiliations:** 1 Department of Cell Biology and Neuroscience, Rutgers University, Piscataway, NJ 08854, USA; 2 Stem Cell Research Center and Keck Center for Collaborative Neuroscience, Rutgers University, Piscataway, NJ 08854, USA

**Keywords:** PI3 kinase, Aminoglycoside, Hair cell, Ototoxicity, Prevention

## Abstract

Loss of sensory hair cells of the inner ear due to aminoglycoside exposure is a major cause of hearing loss. Using an immortalized multipotent otic progenitor (iMOP) cell line, specific signaling pathways that promote otic cell survival were identified. Of the signaling pathways identified, the PI3K pathway emerged as a strong candidate for promoting hair cell survival. In aging animals, components for active PI3K signaling are present but decrease in hair cells. In this study, we determined whether activated PI3K signaling in hair cells promotes survival. To activate PI3K signaling in hair cells, we used a small molecule inhibitor of PTEN or genetically ablated PTEN using a conditional knockout animal. Hair cell survival was challenged by addition of gentamicin to cochlear cultures. Hair cells with activated PI3K signaling were more resistant to aminoglycoside-induced hair cell death. These results indicate that increased PI3K signaling in hair cells promote survival and the PI3K signaling pathway is a target for preventing aminoglycoside-induced hearing loss.

## Abstract (precis)

**Summary:** Activation of PI3K signaling using either a pharmacological inhibitor or genetic ablation of PTEN protects inner ear hair cells from aminoglycoside-induced death.

## INTRODUCTION

Hair cells in the cochlea are the mechanosensitive sensory cells responsible for converting sounds into auditory neural signal. The gradual loss of hair cells contributes to hearing loss. Both environmental and genetic factors contribute to progressive hair cell loss (Jennings and Jones, 2001). Maintenance of hair cell homeostasis and other protective mechanisms play a role in preserving hair cells in aging individuals. A major environmental contributor to hearing loss is the employment of ototoxic drugs. Aminoglycosides are a well-established and successful class of antibiotics used for managing microbial infections. Although commonly used in treating infections, aminoglycosides have devastating ototoxic effects that lead to hearing loss (Huth et al., 2011). Systemic administration and tracing of fluorescently labeled gentamicin, a class of aminoglycosides, suggests specific accumulation within the sensory hair cells of the inner ear (Wang and Steyger, 2009). These results are consistent with gentamicin accumulation in hair cells across different animal species (Imamura and Adams, 2003). Gentamicin either enters through the hair cell transduction channel or is endocytosed via the apical surface of the hair cell resulting in intracellular accumulation (Alharazneh et al., 2011; Tran Ba Huy et al., 1986; Wang and Steyger, 2009). Once in the hair cells, gentamicin leads to apoptosis and likely acts through multiple mechanisms including reactive oxygen species (ROS) formation (Huth et al., 2011). The increase in ROS generates toxic free radicals (Clerici et al., 1996; Hirose et al., 1997; Sha and Schacht, 1999a,b). When cellular protective mechanisms are overwhelmed in hair cells by the toxic effects of free radicals, hair cells undergo apoptotic cell death (Cheng et al., 2005). To maintain viability of hair cells after ototoxic damage, one strategy is to activate homeostatic and other protective mechanisms to promote hair cell survival.

Many genes are differentially expressed during hair cell development (Hawkins and Lovett, 2004; Ryan, 2002). We hypothesize that genes expressed during hair cell differentiation may be important for maintaining hair cell survival. Using immortalized multipotent otic progenitor (iMOP) cells as an *in vitro* cellular system for otic development, we sought to identify genes that could be responsible for maintaining hair cell survival. iMOP cells are a fate-restricted cell type generated from embryonic neurosensory precursors and immortalized by transient C-MYC expression. iMOP cells continually self-renew but retain the capacity to differentiate into functional hair cells and supporting cells under the appropriate conditions (Kwan et al., 2015). In addition, transcripts associated with hair cells (MYO6) and supporting cells (TECTA and OTOA) are upregulated during iMOP differentiation, which further suggests their validity as a cellular model for these inner ear cell types (Kwan et al., 2015).

## RESULTS

### Differentiating iMOP cells exit the cell cycle and express hair cell and supporting cell markers

**Fig. 1.**
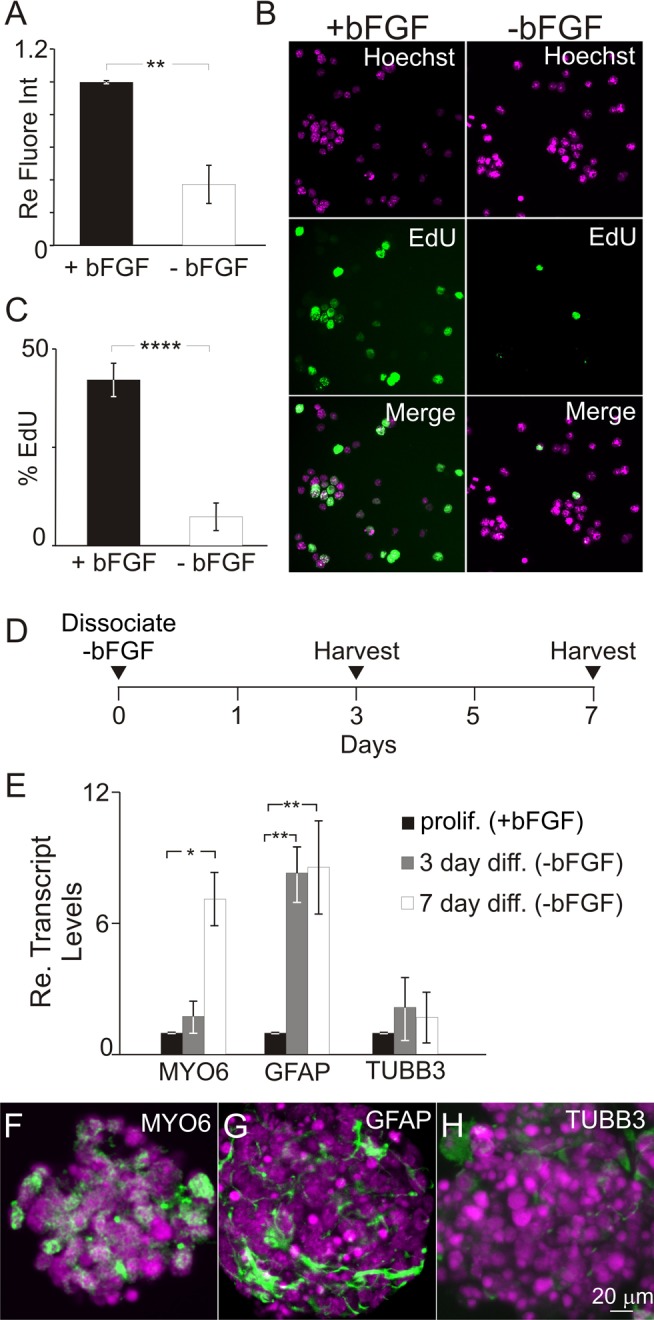
**Proliferation and differentiation potential of iMOP cells after bFGF withdrawal.** (A) Fluorescence intensity from DNA binding dye on iMOP cells cultured in the presence (*n*=3) or absence (*n*=3) of bFGF for 3 days. Fluorescence intensities are normalized to proliferating cultures to reflect changes in cell numbers. (B) Immunofluorescence images of EdU-labeled iMOP cells cultured in the presence or absence of bFGF for 3 days. (C) Percentages of EdU-labeled iMOP cells grown in the presence (*n*=5) or absence (*n*=5) of bFGF for 3 days. (D) Timeline for harvesting iMOP cells after growth factor withdrawal. (E) qPCR depicting relative transcript levels of MYO6, GFAP, and TUBB3 transcripts in proliferating (*n*=3) and differentiating iMOP cells cultured in absence bFGF for 3 days (*n*=3) and 7 days (*n*=3). Transcript levels are normalized to GAPDH and expressed as an average fold-change. (F-H) Representative immunofluorescence images from 7 day-differentiated iMOP cells for (F) MYO6 (*n*=3), (G) GFAP (*n*=3), and (H) TUBB3 (*n*=3). Data represented as mean±s.d.; *P<0.05, **P<1×10^−2^, ****P<1×10^−4^ by unpaired two-tailed Student's *t*-test.

iMOP cultures allow for harvesting of a large number of otic fate restricted cells for RNA-seq. Proliferating iMOP cells were grown in suspension as colony-forming otic cells, known as otospheres. To initiate differentiation into hair cells and supporting cells bFGF, the sole growth factor in the media, was withdrawn from iMOP cultures (Jadali et al., 2015). Two methods were employed to monitor cell cycle arrest. First, a fluorescence-based assay was used as a measure of cell numbers to determine the proliferative status of the cultures. iMOP cells were cultured either in the presence or absence of bFGF for 3 days before labeling with CyQuant direct nucleic acid stain, a cell permeable fluorescent DNA dye to assay for total DNA content. Emitted fluorescence from the DNA bound dye served as an index of total cell numbers. Cultures grown in the absence of bFGF showed a significant decrease in cell numbers compared to proliferating cultures (*P*<1×10^−2^) (Fig. 1A).

Next, to determine whether cells were exiting the cell cycle after bFGF withdrawal, incorporation of the nucleotide analog EdU was used as a measure of cells in S phase. Immunofluorescence images of EdU labeled iMOP cells cultured in the presence or absence of bFGF for 3 days demonstrated a decrease in EdU labeled cells after withdrawal of bFGF (Fig. 1B). Quantification of EdU labeled iMOP cells indicated that 42% of cells incorporated EdU in bFGF cultures, while only 7% of cells in cultures without bFGF incorporated EdU (*P*<1×10^−4^) (Fig. 1C). Together, these results demonstrated that bFGF withdrawal significantly reduced the percentage of cells undergoing an active cell cycle.

**Fig. 2.**
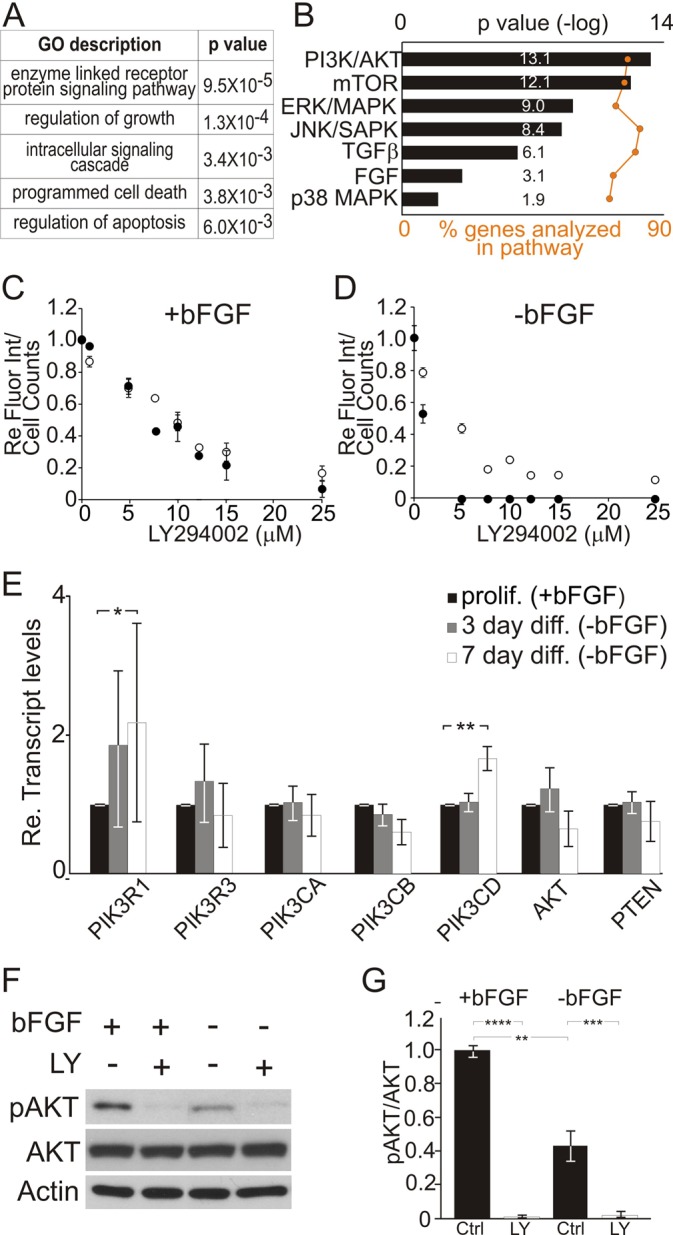
**Gene ontology analysis of iMOP cells.** Differentially expressed genes from proliferating and differentiating iMOP cells have been identified from RNA-seq data for analysis. (A) Predicted biological processes for differentially expressed genes using the Database for Annotation, Visualization and Integrated Discovery (DAVID) are shown. The gene ontology (GO) terms for biological processes are listed along with their *P*-values. (B) Signal transduction pathways identified from canonical pathway analysis using Ingenuity Pathway Analysis (IPA) software. Top pathways are displayed as bar graphs along with the associated *P*-values. The percentages of genes identified from the RNA-seq data that correspond to pre-defined genes in each canonical pathway are shown as data points along an orange line. (C,D) Fluorescence-based cell number assay (black) and cell counts (white) from (C) proliferating iMOP cultures and (D) differentiating iMOP cells treated with a various concentrations of LY294002 are shown (*n*=4). (E) qPCR of genes in the PI3K signaling pathway (PIK3R1, PIK3R3, PIK3CA, PIK3CB, PIK3CD, AKT and PTEN) in proliferating (*n*=3) and differentiating iMOP cells cultured in absence of growth factor for 3 days (*n*=3) or 7 days (*n*=3). Transcript levels are normalized to GAPDH and expressed as relative fold increase. (F) Western blot of phospho-AKT and total AKT in proliferating and differentiating iMOP cells cultured in the presence or absence of 25 µM LY294002 (LY) for 1 day. Actin bands are used as loading controls. (G) Quantification of phospho-AKT/total AKT in proliferating (*n*=3) and differentiating (*n*=3) iMOP cells cultured in the presence (*n*=3) or absence (*n*=3) of 25 µM LY294002 for 1 day. Data represented as mean±s.d.; *P<0.05, **P<1×10^−2^, ***P<1×10^−3^, ****P<1×10^−4^ by unpaired two-tailed Student's *t*-test.

When cultured as otospheres, cell cycle exit promotes differentiation of iMOP cells into hair cells and supporting cells (Jadali et al., 2015; Kwan et al., 2015). To assess whether iMOP cells have initiated differentiation after growth factor withdrawal, transcript levels of MYO6, GFAP and TUBB3 were determined by quantitative real-time PCR (qPCR). MYO6 was used as a hair cell marker, while GFAP and TUBB3 expression were used as supporting cell and neuronal markers. iMOP cells were grown in the presence of bFGF for 3 days and in the absence of bFGF for 3 or 7 days before harvesting (Fig. 1D). Comparison of transcripts from these samples represented snapshots of iMOP cells undergoing otic differentiation. After removal of bFGF, iMOP cells displayed a gradual and significant increase in MYO6 transcripts 7 days after bFGF withdrawal (*P*<0.05) compared to cultures grown in the presence of bFGF (Fig. 1E). GFAP transcript levels significantly increased within 3 days of growth factor withdrawal (*P*<1×10^−2^) and remained high after 7 days (Fig. 1E). TUBB3 transcript levels only displayed a slight and not statistically significant increase (Fig. 1E). Immunostaining of 7 day differentiated iMOP cultures indicated expression of MYO6 (Fig. 2F), GFAP (Fig. 2G), and low levels of TUBB3 (Fig. 2H). Together, these data demonstrated that iMOP cells exit the cell cycle and express hair cell and supporting cell markers that correlate to otic differentiation. From hereafter, we refer to cells cultured in presence of bFGF as proliferating iMOP cells and cells cultured in the absence of bFGF as differentiating iMOP cells.

### Identification of signaling pathways involved in iMOP cell survival

To better understand the cellular process underlying iMOP cells during proliferation and differentiation, we compared the transcriptome between proliferating and differentiating iMOP cells. RNA-seq was performed on iMOP cells cultured in the presence or absence of bFGF for 7 days. Biological replicates from RNA-seq samples were averaged and used to perform a pairwise comparison between the two culture conditions. Proliferating and differentiating iMOP cells displayed 5103 significantly altered transcripts (*P*<0.05). To predict the cellular processes, differentially expressed genes were subjected to gene ontology analysis using the Database for Annotation, Visualization and Integrated Discovery (DAVID) (Huang et al., 2009a,b). Cellular processes that include signal transduction, cell growth and regulation of apoptosis were identified (Fig. 2A). From these results, we hypothesized that signaling pathways contribute to these cellular processes such as regulation of proliferation and cell survival.

To determine the specific signaling pathways represented by differentially expressed genes, a canonical pathway analysis using Ingenuity Pathway Analysis (IPA) software was performed. For each of the signaling pathways described, ∼85% of known genes in these pathways were detected in the RNA-seq and used for analysis (Fig. 2B). A large number of transcripts corresponded to PI3K/AKT, JNK, MAPK and mTOR signaling pathways. The identified pathways were predicted to be downregulated but sustained during iMOP differentiation. These results predict an important role for these signaling pathways in proliferation or survival of iMOP cells.

### Validating signaling pathways using small molecule inhibitors

To determine how signaling pathways affect cellular function, small molecule inhibitors were used to determine the effects on proliferation and cell survival in iMOP cells. LY294002 was used to inhibit the PI3K pathway (Vlahos et al., 1994), SP600125 to inhibit the JNK pathway (Bennett et al., 2001), U0126 to inhibit the MAPK pathway (Favata et al., 1998) and rapamycin to inhibit mTOR signaling (Chung et al., 1992; Price et al., 1992). The concentration range of inhibitors used was initially established using published results from other cell lines. Proliferating and differentiating iMOP cells were treated with 1-25 µM LY294002 (Fig. 2C,D), 1-25 µM SP600125 (Fig. S1A,B), 5-40 µM U0126 (Fig. S1C,D) and 1-25 nM rapamycin (Fig. S1E,F), and assayed using a fluorescence-based assay as an index of cell numbers. In parallel, the cell numbers from these cultures were determined using a cell counter (Fig. 2C,D; Fig. S1A,B; S1C,D; S1E,F, respectively). Quantitation of the fluorescence intensities and cell numbers were normalized to DMSO controls. The IC-50 for each inhibitor was calculated after curve fitting of data from proliferating and differentiating iMOP cells. The IC-50 for LY294002 was 11 µM for proliferating and 6 µM for differentiating cells; IC-50 for SP600125 was 9 µM for both proliferating and differentiating cells. The IC-50 for U0126 was 21 µM and 20 µM for proliferating and differentiating cells respectively. Inhibition of mTOR signaling using rapamycin only showed a significant reduction in fluorescence intensity and cell counts in differentiating iMOP cells. Subsequently, only the IC-50 in differentiating iMOP cells was calculated. The IC-50 for rapamycin was 17 nM. mTOR does not play a significant role in proliferating cells and most likely plays a role in survival of differentiating cells.

These results suggested that most of the candidate signaling pathways could affect iMOP proliferation or cell survival. Since PI3K signaling was predicted to be the most significant pathway that contributes to the predicted cellular process of proliferation or cell survival (Fig. 2B) and PI3K signaling has been shown to be upstream of MAPK/ERK, JNK and mTOR signaling (Carracedo and Pandolfi, 2008; Steelman et al., 2004), we focused on PI3K signaling. The function of a signaling pathway is dependent on cellular context and can range from proliferation, differentiation or cell survival. PI3K signaling has been described to play an important role during hair cell development (Okano et al., 2011). During inner ear development, PI3K signaling has been implicated in proliferation of otic progenitors (Sun et al., 2014) but its role as a therapeutic target for promoting cell survival has not been explored. To confirm the presence of transcripts in the PI3K signaling pathway as demonstrated in RNA-seq data, qPCR was performed. Transcript levels of PIK3R1, PIK3R3, PIK3CA, PIK3CB, PIK3CD, AKT and PTEN from proliferating iMOP cells were compared to 3- and 7-day differentiating iMOP cells. PIK3R1 and PIK3R3 are distinct isozymes of the regulatory subunit of PI3K and PIK3CA, PIK3CB and PIK3CD are different isozymes of the catalytic subunit of PI3K (Braccini et al., 2012). AKT is the major downstream target of PI3K and PTEN is a phosphatase that modulates phosphotidyl inositol levels at the cell membrane to regulate PI3K signaling (Liang and Slingerland, 2003). qPCR of these genes confirmed the presence of various transcripts in both proliferating and differentiating iMOP cells (Fig. 2E). Furthermore, qPCR data revealed a twofold increase in PI3KR1 (*P*<0.05) and a twofold increase in PI3KCD (*P*<1×10^−2^) expression after 7 days of differentiation (Fig. 2E). Although the presence of transcripts was confirmed by qPCR, changes in transcript levels are not indicative of an active signaling pathway. Therefore, we wanted to determine the activity of the PI3K signaling by looking at phosphorylation of downstream target genes.

AKT is a major downstream target of PI3K signaling (Coffer et al., 1998; Liang and Slingerland, 2003; Manning and Cantley, 2007). To assay for alterations in PI3K signaling, we determined changes in AKT phosphorylation at Thr308 using a phospho-specific antibody in proliferating and differentiating iMOP cells. Levels of phospho-AKT reflect PI3K signaling activity levels (Coffer et al., 1998; Liang and Slingerland, 2003). Western blot analysis demonstrated that removal of bFGF decreased AKT phosphorylation in differentiating iMOP cells relative to proliferating cells (Fig. 2F). Furthermore, treatment with 25 µM LY294002 significantly decreased phospho-AKT relative to total AKT in both proliferating and differentiating iMOP cells (Fig. 2F). To quantify and normalize phospho-AKT levels, the ratio of phospho-AKT/total AKT was determined. Comparison of the phosho-AKT/total AKT ratios in proliferating and differentiating cells demonstrated a twofold decrease after bFGF removal (*P*<1×10^−2^) (Fig. 2G). Additionally, normalized phospho-AKT levels decreased by tenfold in proliferating iMOP cells (*P*<1×10^−4^) and decreased by sixfold in differentiating iMOP cells (*P*<1×10^−3^) after treatment with LY294002 (Fig. 2G). These results confirmed that PI3K signaling was activate in both proliferating and differentiating iMOP cells and could be inhibited using LY294002.

### Activation of PI3K promotes iMOP cell survival

**Fig. 3.**
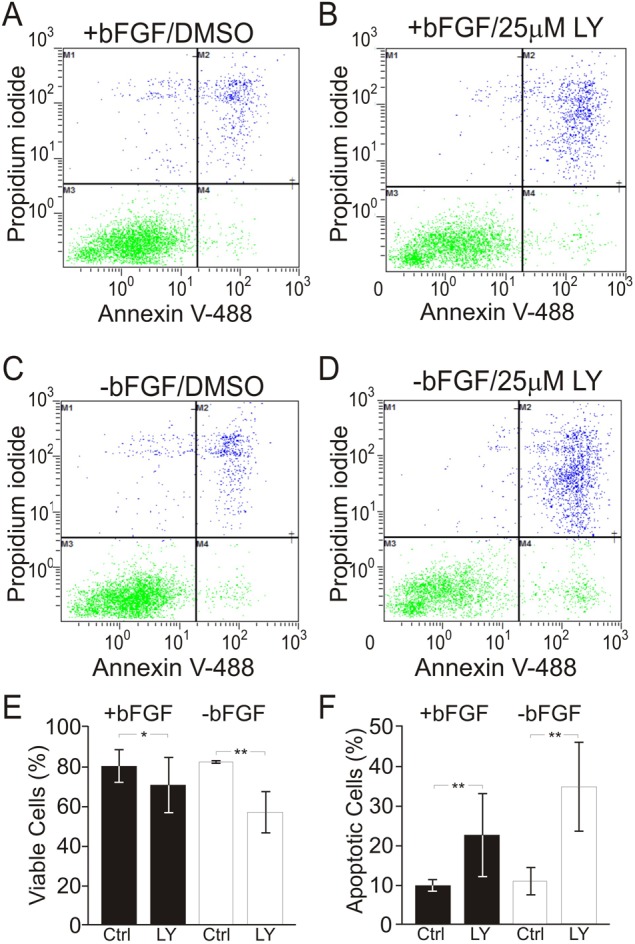
**Effects of PI3K inhibition on cell survival in iMOP cells.** (A,B) Representative FACS plot of PI- and annexin V-labeled iMOP cells cultured in the presence of bFGF and treated with (A) DMSO as control (*n*=3) or (B) 25 µM LY294002 (LY; *n*=3). (C,D) Representative FACS plot for iMOP cells cultured in the absence of bFGF for 3 days and treated with (C) DMSO as control (*n*=3) or (D) 25 µM LY294002 (*n*=3). (E) Percentages of viable cells (PI− annexinV−) from FACS data in control and LY294002-treated cells cultured in the presence or absence of bFGF. (F) Percentages of apoptotic iMOP cells (PI+ annexinV+) from FACS data for control and LY294002 iMOP cultures grown in the presence or absence of bFGF. Data represented as mean±s.d.; *P<0.05, **P<1×10^−2^ by unpaired two-tailed Student's *t*-test.

Treatment of iMOP cells with LY294002 suggested a potential role for PI3K signaling in proliferation or survival. To determine whether PI3K signaling affected cell survival, proliferating and differentiating iMOP cells were treated with 25 µM LY294002 to inhibit PI3K signaling. Inhibition of PI3K signaling would increase the number of apoptotic cells if the signaling pathway was important for cell survival. To assay for apoptosis, cells were dually labeled with propidium iodide (PI) and annexin V (Vermes et al., 1995). Labeled cells were subjected to flow cytometry and the percentages of differentially labeled cells determined. To determine the effects of LY294002, unlabeled viable cells and dually labeled PI and annexin V apoptotic cells were analyzed. Proliferating iMOP cultures treated with DMSO (Fig. 3A) or 25 µM LY294002 (Fig. 3B) were subjected to FACS analysis. Similarly, differentiating iMOP cultures were treated with DMSO (Fig. 3C) or 25 µM LY294002 (Fig. 3D). After LY294002 treatment, the percentage of viable cells decreased from 80% to 65% (*P*<0.05) in proliferating cells and from 82% to 57% (*P*<1×10^−2^) in differentiating cells (Fig. 3E). Concurrent to the decrease in viable cells, the percentage of apoptotic cells in proliferating iMOP cells increased from 10% to 23% (*P*<1×10^−2^) and from 11% to 35% (*P*<1×10^−2^) in differentiating iMOP cells (Fig. 3F). These results demonstrated that inhibition of the PI3K pathway with LY294002 decreased cell survival. In proliferating iMOP cells, inhibiting PI3K signaling could affect both proliferation and cell survival. In contrast, during differentiation, the vast majority of the cells are no longer dividing and the main role of PI3K is to promote cell survival. We propose that the main role of PI3K signaling in post-mitotic hair cells is to maintain cell survival.

**Fig. 4.**
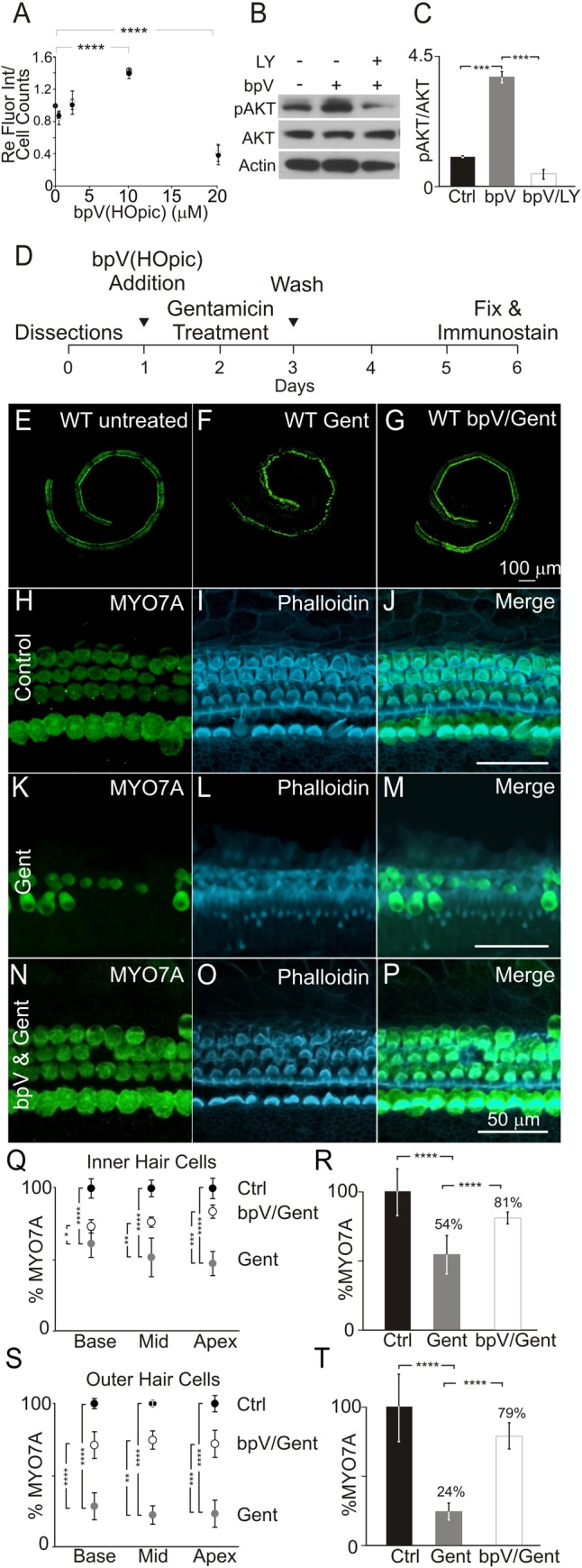
**Effect of bpV(HOpic) on hair cell survival.** Differentiating iMOP cells were cultured with a range of bpV(HOpic) concentrations to determine effects on cell survival. (A) Relative fluorescence intensity as a measure of changes in cell numbers after treatment with different concentrations of bpV(HOpic) (*n*=3). (B) Western blot of phospho-AKT and total AKT from control, 10 µM bpV(HOpic) treated (bpV) and 10 µM bpV(HOpic)/25 µM LY294002 (LY) treated iMOP cultures. Actin serves as a loading control. (C) Ratio of phospho-AKT/total AKT from control (*n*=3), bpV(HOpic)-treated (*n*=3), and bpV(HOpic)/LY294002 treated (*n*=3) cultures. (D) Timeline of cochlear explant cultures describing the addition of 10 µM bpV(HOpic) 1 day before 50 µM gentamicin treatment. (E-G) Representative stitched fluorescence image MYO7A-labeled hair cells in (E) control (*n*=4), (F) 50 µM gentamicin treated (*n*=4), and (G) 10 µM bpV(HOpic)/50 µM gentamicin treated (*n*=4) cochlear explants. (H-P) Immunofluorescence images of MYO7A, phalloidin and merged images in (H-J) control (*n*=4), (K-M) 50 µM gentamicin treated (*n*=4) and (N-P) 10 µM bpV(HOpic)/50 µM gentamicin treated (*n*=4) cochlear explants. (Q) Quantification of MYO7A IHC from the basal, middle and apical regions of cochlea explants. Percentages of IHC in control (black) (*n*=4), gentamicin treated (gray) (*n*=4) and bpv(HOpic)/gentamicin treated (white) (*n*=4) are normalized to control. (R) Percentage of MYO7A IHC from control (black; *n*=4), gentamicin-treated (gray; *n*=4) and bpV(HOpic)/gentamicin-treated cultures (white; *n*=4). (S) Percentage of MYO7A OHC numbers in control (black; *n*=4), gentamicin-treated (gray; *n*=4) and bpv(HOpic)/gentamicin-treated (white; *n*=4) cultures from the basal, middle, and apical regions of cochlear explants. (T) Total percentage of MYO7A labeled OHC from control (black; *n*=4), gentamicin-treated (gray; *n*=4) and bpV(HOpic)/gentamicin-treated cultures (white; *n*=4). Data represented as mean±s.d.; *P<0.05, **P<1×10^−2^, ***P<1×10^−3^, ****P<1×10^−4^ by unpaired two-tailed Student's *t*-test.

In aging mice, protein levels of phosphatase tension homolog deleted on chromosome 10 (PTEN) increases to attenuate PI3K signaling (Sha et al., 2010). PTEN antagonizes the PI3K lipid kinase activity by converting phosphatidylinositol 3,4,5-trisphosphate (PIP_3_) into phosphatidylinositol 4,5-bisphosphate (PIP_2_). Inhibition of PTEN increases PIP_3_ in the cell membrane and activates pathways downstream of PI3K (Cantley and Neel, 1999; Doillon et al., 1999; Schmid et al., 2004). Increased PTEN levels correlates to a declining capacity of hair cells to survive (Sha et al., 2010). Using differentiating iMOP cells to study the effects of PI3K signaling in hair cell survival, we hypothesized that inhibition of PTEN sustains PI3K signaling and could increase cell survival. A small molecule, bpV(HOpic), was used to inhibit PTEN. To determine the optimal concentration of bpV(HOpic) to increase cell survival, differentiating iMOP cells were treated with different concentrations of bpV(HOpic) for 3 days. Cells were then subjected to the fluorescence-based cell number assay and cell counts as a measure of cell survival. Addition of bpV(HOpic) showed a gradual increase in cell numbers up to 10 µM. At 10 µM bpV(HOpic), iMOP cultures showed a 1.5-fold increase in both fluorescence intensity (*P*<1×10^−4^) and total cell counts (*P*<1×10^−4^) relative to controls (Fig. 4A). Incubation with 20 µM bpV(HOpic) resulted in a threefold decrease in both cell numbers (*P*<1×10^−4^) and cell counts (*P*<1×10^−4^). These results suggested that optimal increase in cell survival can be attained using 10 µM bpV(HOpic) and higher concentrations lead to cellular toxicity.

To ensure that bpV(HOpic) activated PI3K signaling, we looked for increases in normalized phospho-AKT levels. Differentiating iMOP cells were treated with 10 µM bpV(HOpic) for 1 day. Western blot analysis demonstrated a significant increase in phospho-AKT after addition of bpV(HOpic) (Fig. 4B). Quantification of phospho-AKT/total AKT ratios showed a fourfold increase in normalized phospho-AKT levels (*P*<1×10^−3^) after 10 µM bpV(HOpic) treatment (Fig. 4C). To confirm that bpV(HOpic) treatment specifically activated PI3K signaling, differentiated iMOP cells were pre-treated with 10 µM bpV(HOpic) for 1 h before inhibiting PI3K signaling using 25 µM LY294002 for 1 day. In contrast to bpV(HOpic) treated samples, samples treated with both LY294002 and bpV(HOpic) showed a fourfold reduction in phospho-AKT levels (*P*<1×10^−3^) (Fig. 4C). These results demonstrated that addition of bpV(HOpic) increased PI3K signaling in differentiating iMOP cells.

### Sustained PI3K signaling using bpV(HOpic) prevents gentamicin-induced hair cell death

Since activated PI3K signaling enhanced survival of differentiating iMOP cells, we reasoned that it may also increase survival of hair cells. To challenge hair cell survival, murine cochlear explants were exposed to gentamicin. Cochleae were obtained from post-natal mice, explanted, and allowed to recover for 24 h after dissections. Based on the dose response curve with bpV(HOpic) in iMOP cells, cochlear explants were treated with 10 µM bpV(HOpic). One day after addition of bpV(HOpic), cochleae were exposed to 50 µM of gentamicin for 24 h before the cultures were washed with Hank's Balanced Salt Solution (HBSS) and replaced with fresh media without gentamicin or bpV(HOpic). Cochlear explants were allowed to recover for 3 days, fixed, and subjected to immunostaining (Fig. 4D). Hair cell counts were used to determine the effects of bpV(HOpic) on hair cell survival. To label the hair cells, cochleae were immmunostained for MYO7A to label the cell bodies and phalloidin was used to highlight the actin filament filled hair bundles. In control cochlear explants, MYO7A-positive staining of inner hair cells (IHC) and outer hair cells (OHC) (green) can be observed throughout the length of the cochlea (Fig. 4E). Similar immunostaining with cochlear explants were accomplished after treatment with 50 µM gentamicin (Fig. 4F) or 10 µM bpV(HOpic)/50 µM gentamicin (Fig. 4G).

To quantify the number of IHC and OHC in the cochlea, MYO7A labeled cell bodies were counted and phalloidin was used as a qualitative measure of hair bundle integrity. Compared to controls (Fig. 4H-J), treatment of cochlear cultures with 50 µM gentamicin for 24 h resulted in a significant loss MYO7A and phalloidin labeled hair cells in the sensory epithelia (Fig. 4K-M). Pre-treatment with 10 µM bpV(HOpic) significantly improved the survival of hair cells and retained MYO7A labeled hair cells with phalloidin marked hair bundles (Fig. 4N-P). Since gentamicin-induced toxicity could have different effects on hair cells along the length of the cochlea and could affect IHC and OHC differently, we quantified the two hair cell types separately depending on their location along the cochlea.

To determine the tonotopic counts, the length of the cochlear explant was divided into three equal sections to represent the basal, middle and apical portions of the cochlea. MYO7A positive IHC from these regions were separately quantified and normalized to controls. The percent of IHC in controls were compared to the percent of IHC in gentamicin treated and bpV(HOpic)/gentamicin treated cochlear explants. After gentamicin treatment, a significant decrease in the percentage of IHC from 100% to 62% in the base (*P*<1×10^−4^), 100% to 52% in the middle (*P*<1×10^−4^), and 100% to 48% in the apex (*P*<1×10^−4^) was observed. Pre-treatment with 10 µM bpV(HOpic) before addition of gentamicin resulted in a substantial increase in the percentage of IHC from 62% to 74% in the base (*P*<0.05), 52% to 77% in the middle (*P*<1×10^−2^), and 48% to 84% in the apex (*P*<0.001) (Fig. 4Q). Combining the total percentage of IHC along the length of the cochlea, treatment with gentamicin decreased IHC from 100% to 54% (*P*<1×10^−4^), while pre-treatment with bpV(HOpic) retained hair cells and increased the percentage of surviving IHC from 54% to 81% (*P*<1×10^−4^) (Fig. 4R).

Tonotopic OHC counts comparing the percentage of OHC in control to 50 µM gentamicin treated cochlea showed a significant decrease in the percentage of OHC from 100% to 28% in the base (*P*<1×10^−4^), 100% to 22% in the middle (*P*<1×10^−4^), and 100% to 23% in the apex (*P*<1×10^−4^). Addition of 10 µM bpV(HOpic) before exposure to gentamicin significantly increased the percentage of surviving OHC from 28% to 71% in the base (*P*<1×10^−4^), 22% to 75% in the middle (*P*<1×10^−4^), and 23% to 72% in the apex (*P*<1×10^−4^) (Fig. 4S). Combining the total percentage of OHC along the length of the cochlea, treatment with gentamicin decreased the percentage of in OHC from 100% to 24% (*P*<1×10^−4^). Pre-treatment with bpV(HOpic) before addition of gentamicin prevented hair cell loss and increased the percentage of OHC from 24% to 79% (*P*<1×10^−4^) (Fig. 4T). These results indicated that gentamicin-induced hair cell loss was more severe in OHC than IHC and the addition of bpV(HOpic) significantly prevented gentamicin-induced loss of both hair cell types.

### Activation of PI3K pathway using PTEN conditional knockout mice prevents gentamicin-induced hair cell death

**Fig. 5.**
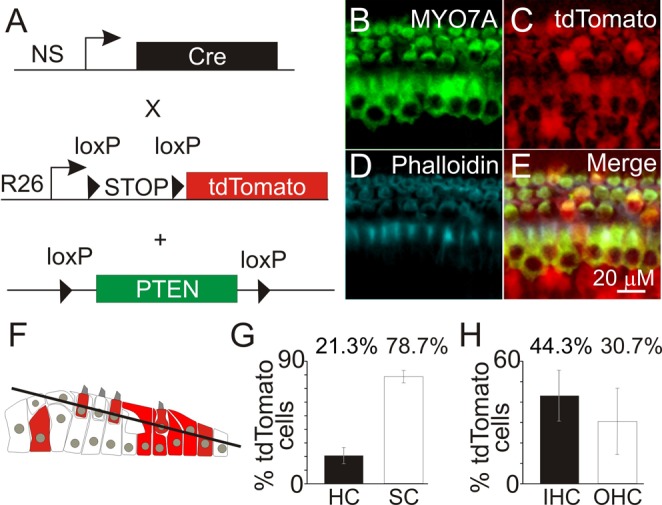
**Ablation and visualization of PTEN knockout cochlear cells.** (A) Diagram of the neural subset (NS) Cre recombinase transgene, a STOP-floxed tdTomato reporter and a PTEN conditional knockout allele in the PTEN cKO mouse line. (B-D) Immunofluorescence images for (B) MYO7A-, (C) tdTomato- and (D) phalloidin-labeled hair cells from the NS Cre tdTomato PTEN conditional allele (PTEN cKO) mouse. (E) Merged image of MYO7A-, tdTomato- and phalloidin-labeled cells for hair cell counts. (F) Diagram of cells in the sensory epithelia. Red cells represent tdTomato-expressing cells. Line represents an optical section acquired by confocal microscopy. (G) Percentage of tdTomato-expressing hair cells (HC) or surrounding cells (SC) (*n*=4) (H) Percentage of tdTomato-expressing IHC and OHC (*n*=4). Data represented as mean±s.d.

To validate the use of bpV(HOpic) for activating PI3K signaling, we employed a PTEN conditional knockout mouse model. Since bpV(HOpic) in the cochlear explants could affect both hair cell and supporting cell types, we used a neural-subset (NS) Cre recombinase mouse (Ljungberg et al., 2009) that targets both hair cells and supporting cells for Cre-mediated PTEN excision. The NS Cre line was generated using a fragment of the human GFAP promoter to drive Cre recombinase (Backman et al., 2001). To visualize and correlate Cre expression in the cochlea, the NS Cre mouse was mated to a red fluorescent protein (tdTomato) reporter mouse (Madisen et al., 2010). In these reporter mice, expression of Cre mediates excision of a loxP-flanked STOP cassette and allows for tdTomato expression. Expression of tdTomato correlates to Cre excision of the PTEN conditional knockout allele resulting in the loss of PTEN (Fig. 5A). We generated a NS Cre tdTomato PTEN conditional knockout mouse (PTEN cKO) to study the effects of upregulating PI3K signaling in the cochlea.

To determine the distribution of PTEN deleted cells in the cochlear sensory epithelium, we visualized the cell types expressing the tdTomato reporter in the PTEN cKO cochlea. To determine the percentage of hair cells that express tdTomato, we immmunostained cochleae obtained from PTEN cKO animals with MYO7A and phalloidin (Fig. 5B-E). Confocal microscopy was used to obtain optical sections from immmunostained cochlea to identify hair cells and tdTomato cells (Fig. 5F). Quantification of tdTomato labeled hair cells relative to all MYO7A and phalloidin labeled hair cells indicated that 21.3% of tdTomato cells were hair cells and the remaining 78.6% were surrounding cells (Fig. 5G). Of the total number of hair cells, 44% of IHC and 31% of OHC expressed tdTomato (Fig. 5H). These data show that within the sensory epithelia, both wild-type and PTEN knockout hair cells and surrounding cells are present. The mosaic expression of the NS Cre reporter animals provides a unique opportunity to compare the effects of activating PI3K signaling in hair cells and surrounding cells to promote hair cell survival during aminoglycoside-induced hair cell death.

**Fig. 6.**
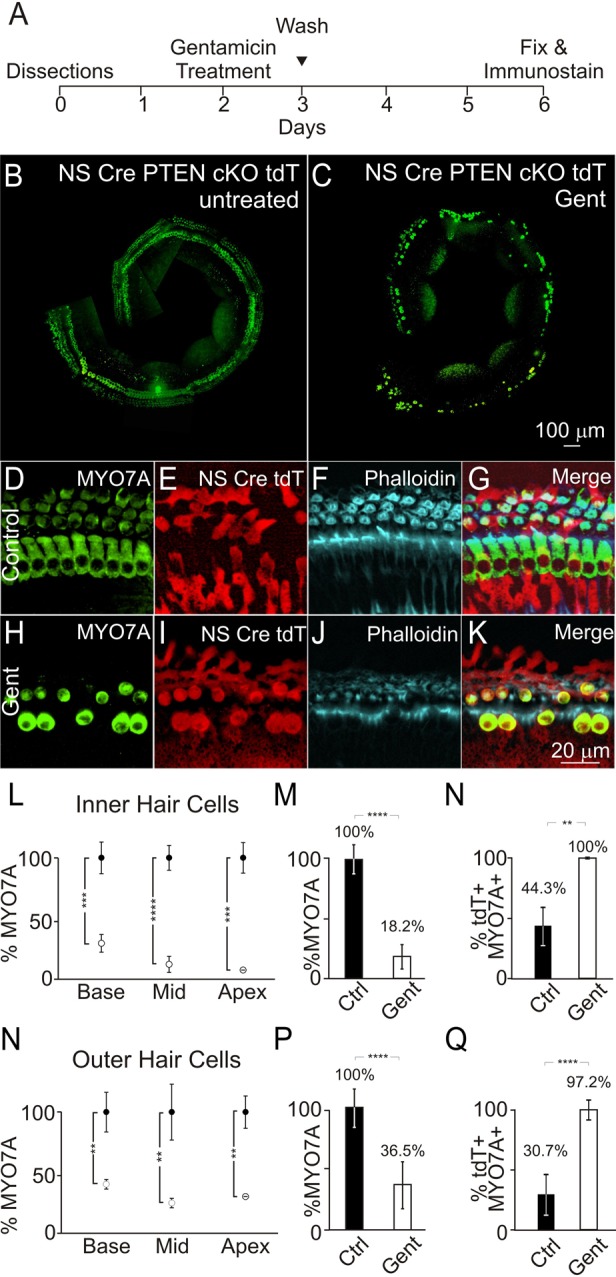
**Hair cell survival after gentamicin-induced damage in PTEN conditional knockout cochlea.** (A) Timeline for treatment of PTEN cKO cochlea explants cultures with gentamicin. (B,C) Representative stitched fluorescence images of MYO7A-labeled hair cells from PTEN cKO cochlear explants in (B) control (*n*=4) and (C) 50 µM gentamicin-treated (*n*=4) samples. (D-F) Fluorescence images of (D) MYO7A-, (E) tdTomato- and (F) phalloidin-labeled cochlea from untreated PTEN cKO animals (*n*=4). (G) Merged image of MYO7A, tdTomato and phalloidin labeling used for hair cell counts. (H-J) Fluorescence images from gentamicin-treated cochlea labeled with (H) MYO7A, (I) tdTomato and (J) phalloidin are shown. (K) Merged image of MYO7A, tdTomato and phalloidin after gentamicin damage for hair cell counts. (L) Percentages of MYO7A IHC in the basal, middle and apical region of the cochlea from untreated (black; *n*=4) and gentamicin-treated (white; *n*=4) cochlea are depicted. (M) Total percentage of MYO7A IHC in untreated (black; *n*=4) and gentamicin treated (white; *n*=4) cochlear explants. (N) MYO7A- and tdTomato-labeled IHC from control (black; *n*=4) and gentamicin treated (white; *n*=4) IHC. (O) Percentage of OHC in the basal, middle, and apical region of the cochlea in control (black; *n*=4) and gentamicin-treated (white; *n*=4) cochleae. (P) Total percentage of MYO7A OHC from control (black; *n*=4) and gentamicin treated (white; *n*=4) cochlea. (Q) MYO7A and tdTomato OHC in untreated (black; *n*=4) and gentamicin-treated (white; *n*=4) cultures. Data represented as mean±s.d.; **P<1×10^−2^, ***P<1×10^−3^, ****P<1×10^−4^ by unpaired two-tailed Student's *t*-test.

To induce ototoxic damage, cochlear explants from PTEN cKO animals were treated with 50 µM gentamicin 48 h after plating. 24 h after treatment, gentamicin was removed, the cochleae were washed and fresh media was added. Explants were allowed to recover for an additional 3 days before fixation and immunostaining with hair cell markers (Fig. 6A). Cochlear explants were immunostained for MYO7A to mark hair cells along the length of the cochlea in both the control and gentamicin treated cochleae (Fig. 6B,C). Magnified images from untreated control PTEN cKO animals showed labeling of MYO7A and tdTomato labeled hair cells that had hair bundles (Fig. 6D-G). Gentamicin treated cochlea showed a loss of MYO7A labeled hair cells and damaged hair bundles (Fig. 6H-K). To determine the extent of hair cell death caused by gentamicin, quantification of MYO7A labeled IHC and OHC counts along the basal, middle, and apical regions of the cochlea was done. Treatment of explants with gentamicin resulted in a significant loss in the percentage of IHC from 100% to 31% in the base (*P*<1×10^−3^), 100% to 12% in the middle (*P*<1×10^−4^), and 100% to 7% in the apex (*P*<1×10^−3^) (Fig. 6L). Combining the tonotopic counts, we observed a decrease in the percentage of IHC from 100% to 18% after treatment with 50 µM gentamicin (*P*<1×10^−4^) (Fig. 6M).

If activation of PI3K provided an advantage for hair cells to survive ototoxic damage, an increase in the percentage of tdTomato expressing hair cells relative to unlabeled hair cells will be observed. To determine the effects of gentamicin treatment, surviving IHC expressing tdTomato from gentamicin treated cultures were counted. In untreated NS Cre PTEN cKO cochleae, wild-type MYO7A labeled cells that do not express tdTomato constitute 56% of IHC while the remaining 44% IHC are MYO7A/tdTomato. After gentamicin treatment, no wild-type MYO7A labeled hair cells are found. Instead, 100% of the IHCs were marked by MYO7A/tdTomato (*P*<1×10^−2^) (Fig. 6N). These results suggested that PTEN deletion increased survival of IHCs compared to wild-type IHC within the same cochlea.

Similarly, gentamicin treated PTEN cKO explants demonstrated a significant loss in the percentage of OHC from 100% to 40% in the base (*P*<1×10^−2^), 100% to 24% in the middle (*P*<1×10^−2^), and 100% to 29% in the apex (*P*<1×10^−2^) (Fig. 6O) compared to the untreated controls. Combining the percentage of OHC along the length of the cochlea, we observed a loss of total OHC from 100% to 37% after gentamicin treatment (*P*<1×10^−4^) (Fig. 6P). In untreated cultures 69% of OHCs are singly labeled with MYO7A while the remaining 31% of OHC are MYO7A/tdTomato. In gentamicin treated explants, 97% of surviving OHC express tdTomato with almost no remaining wild-type hair cells within the same cochlea (*P*<1×10^−4^) (Fig. 6Q). The remaining 3% of surviving OHCs were surrounded by tdTomato supporting cells. These results demonstrated that PTEN deletion and subsequent activation of PI3K signaling increased hair cell survival in response to gentamicin induced damage. Our data suggested that increased PI3K signaling promotes hair cell survival after exposure to aminoglycosides.

## DISCUSSION

### Identification of signaling pathways involved in hair cell survival

We employed otic fate-restricted iMOP cells to identify important signaling pathways for hair cell survival. We showed that PI3K signaling is an important pathway that affects iMOP cell survival. During development, ablation of PTEN and subsequent activation of PI3K signaling was reported to increase proliferation of otic progenitors and result in increased hair cell numbers (Sun et al., 2014). In addition to the role PI3K signaling plays in proliferation, we showed that activation of PI3K promotes hair cell survival and could play a role in maintaining the viability of hair cells.

Specific components of the PI3K signaling pathway revealed an upregulation of PIK3R1 and PIK3CD during iMOP cell differentiation. An autosomal dominant mutation in the PIK3R1 gene in patients with SHORT syndrome was previously identified (Dyment et al., 2013). SHORT syndrome is a multisystemic disease characterized by Short stature, Hyperextensibility of joints, Ocular depression, Rieger anomaly, and Teething delay (Cohn et al., 1975). In addition to these characteristics, patients with SHORT syndrome show fairly normal auditory brainstem responses (ABRs) at birth. After 1 year of age, over 25% of children show hearing deficits as assayed by ABRs (Joo et al., 1999; Toriello et al., 1985). PIK3R1 (p85α) is the non-catalytic regulatory subunit of the PI3K holoenzyme that is essential for phosphorylating and activating downstream targets (Liang and Slingerland, 2003; Vanhaesebroeck et al., 2012). We propose that PI3K signaling actively maintains survival of cochlear hair cells and loss of PI3K signaling would result in delayed onset of hearing loss. In addition to the crucial role that PI3K signaling plays in promoting hair cell survival, we also identified a potential role for ERK, JNK and mTOR signaling in cell survival. These pathways could act in conjunction or downstream of PI3K signaling to further enhance hair cell survival.

### Activated PI3K signaling prevents aminoglycoside-induced hair cell death

We showed that PI3K signaling plays a major role in survival of iMOP cells and reasoned that the pathway can be repurposed to promote hair cell survival. To activate PI3K signaling, hair cells treated with bpV(HOpic) or derived from PTEN cKO animals were challenged with gentamicin. We showed that activated PI3K signaling increased hair cell survival after ototoxic damage. Previous studies report that in aging mice, loss of hair cells occurs in a base to apex gradient *in vivo* (Plontke et al., 2007; Shone et al., 1991). However, our cochlear cultures did not reveal a base to apex gradient in hair cell loss when treated with gentamicin. These results suggest that age-related hearing loss and aminoglycoside-induced hair cell loss may occur through different cellular mechanisms or that the experimental paradigms used to measure hair cell survival are not directly comparable.

Similar to other small molecules, bpV(HOpic) could promote cell survival by inhibiting other target molecules. To ensure that activation of PI3K signaling by bpV(HOpic) is the responsible for cell survival, a genetic mouse model was used. Ablation of PTEN was accomplished to activate the PI3K signaling pathway. In the NS Cre PTEN knockout cochlea there is a mosaic of PTEN knockout and wild-type hair cells. The vast majority of hair cells that survived gentamicin damage were PTEN nulls and upregulated PI3K signaling. However, a small percentage of surviving wild-type hair cells was surrounded by PTEN knockout supporting cells after ototoxic damage. Supporting cells play a role in development and maintenance of hair cells *in vivo* (May et al., 2013; Mellado Lagarde et al., 2014; Monzack and Cunningham, 2013). In addition to the cell autonomous effects of increased PI3K signaling, we propose that upregulation of PI3K in supporting cells may provide additional intracellular signaling to indirectly promote hair cell survival. There could be two distinct mechanisms to promote hair cell survival after aminoglycoside-induced damage. Activation of PI3K signaling may function in a cell autonomous manner by directly promoting hair cell survival or indirectly mediate hair cell survival through cell-cell interactions with supporting cells.

### PI3K signaling has multiple roles including maintenance of hair cell viability

The PI3K signaling pathway has been studied in many different cell types and has been shown to play a role in proliferation, survival, differentiation, and metabolism in a cell-type dependent manner (Carracedo and Pandolfi, 2008). Treatment of MEFs with LY294002 has been shown to prevent chemotherapy-induced apoptosis (Bar et al., 2005). In primary mouse keratinocytes LY294002 has no effect on proliferating or differentiating cells (Jadali and Ghazizadeh, 2010). In prostate cancer, deletion of PTEN results in aggressive metastatic potential due to increased proliferation (Phin et al., 2013). PTEN loss in hematopoetic stem cells leads to the exhaustion of the stem cell pool (Yilmaz et al., 2006; Zhang et al., 2006). In cortical neurons, ablation of PTEN using the NS Cre Pten cKO mice results in increased cell size (Ljungberg et al., 2009). PTEN deletion in neurons also showed a disruption in neuronal migration and laminar organization in addition to increased soma size (Fraser et al., 2004; Kwon et al., 2001). These studies suggest that PI3K signaling has different cellular functions in different cell types.

In the inner ear, deletion of PTEN resulted in supernumerary hair cells that developed proper hair bundles (Dong et al., 2010a) presumably due to proliferation of hair cell progenitors (Sun et al., 2014). In contrast, ablation of PTEN showed defects in spiral ganglion neuron fasciculation that may lead to apoptosis (Dong et al., 2010b; Kim et al., 2013). PI3K signaling has not been well studied in the aging or damaged cochlea. By increasing PI3K signaling, our results showed increased survival capability of hair cells. In aging mice, PIP_3_ and phospho-AKT levels decrease as a result of increased PTEN levels in the cochlea. The decrease in PI3K signaling correlated with the reduction in survival capability of hair cells in aging organisms (Sha et al., 2010).

Our results fit well with the notion that PI3K signaling plays an active role in maintaining viability of hair cells throughout the lifetime of an organism. This proposal also fits with the finding that inhibiting PI3K signaling may cause hair cell death in early post-natal mouse and rat cochlear explants (Brand et al., 2011; Chung et al., 2006; Haake et al., 2009).

### Potential ligands that activate PI3K signaling to maintain hair cell survival

The presence of proteins involved in active PI3K signaling in mice suggested that extracellular signals are present to activate the pathway (Sha et al., 2010). Activation of PI3K signaling by binding of soluble ligands or chemokines to receptor tyrosine kinases (Chalhoub and Baker, 2009) could be responsible for promoting hair cell survival. Ligands such as insulin-like growth factor (IGF) 1 and IGF2 were reported to maintain hair cell survival (Lou et al., 2015; Malgrange et al., 2002). Mice harboring inactive IGF1 receptors (IGF1R) showed profound sensorineural hearing loss and a delay in hair cell development (Okano et al., 2011). Treatment with soluble factors such as IGF or bFGF was shown to promote hair cell survival (Hayashi et al., 2013; Low et al., 1996).

We propose that soluble factors normally present in the cochlea bind to cell surface receptors, converge on and activate PI3K signaling to maintain hair cell survival. Activation of PI3K signaling increases phospho-AKT levels which in turn prevents apoptosis by degrading the pro-apoptotic Bcl-2 related protein, BAD and preventing p53 dependent transcriptional apoptotic responses (Franke et al., 2003). These findings suggest that the PI3K signaling pathway may actively maintain hair cell viability. Activation of PI3K may be useful in promoting survival of hair cells after aminoglycoside-induced toxicity and the lack of PI3K signaling could be a cause for congenital hearing loss.

## MATERIALS AND METHODS

### Cell culture

iMOP cells were grown in suspension with DMEM/F12 (Life Technologies) containing B27 supplement (Life Technologies), 25 µg/ml carbenecillin and 20 ng/ml bFGF (PeproTech). For differentiation experiments, cells were cultured for 3 days or 7 days in the absence of bFGF. iMOP cells were treated with varying concentrations of U0126, SB203580, rapamycin, LY294002 (LC Laboratories) and bpV(HOpic) (Santa Cruz Biotechnology, Inc.). All chemicals were solubilized in DMSO and added to the media at the described concentrations.

### EdU incorporation assays and quantification of cell numbers

For EdU labeling index the Click-iT EdU Alexa Fluor 488 assay kit (Life Technologies) was used. iMOP cells were pulsed with 1 µM EdU for 2 h. After EdU incorporation, cells were removed from culture, dissociated to generate single cells, fixed, labeled with Alexa Fluor 488 by click chemistry for flourescence labeling of nuclei and mounted on a slide. Flourescence images of labeled cells were taken using epifluorescence microscopy and the percentage of EdU-positive cells in 1000 nuclei was determined. To assay for total cell numbers, a fluorescence CyQUANT DNA dye (Life Technologies) was used. Cells were treated with detection reagent for 1 h at 37°C, dissociated, and plated in 96 well clear bottom plates (Costar) and imaged using the green filter of the Cytofluor multi-well plate reader (PerSeptive Biosystems). In parallel, cell numbers were determined using a Moxi Cell Counter using the Type S cassettes.

### Gene ontology and pathway analysis

RNA-seq data from proliferating iMOP cells and iMOP cells cultured in the absence of bFGF from two independent iMOP samples for each culture condition were used for gene ontology and pathway analysis. RNA-seq data were obtained from GEO under accession GSE62514. Read alignment was accomplished with Tophat in the Tuxedo software package (Bioconductor) using mm9 as a reference sequence. Fastq files from the aligned sequencing data were converted into indexed BAM files using SAMTools. To correlate read counts and differential gene expression, the BAM files were subjected to analysis using CummeRbund. Normalized FPKM from replicates were extracted from CummeRbund output files using the fpkmMatrix command. Matrix containing the Gene ID and FPKM from iMOP cells cultured in the presence or absence of bFGF identified. The list of genes containing the Gene ID and averaged FPKM were imported into Ingenuity Pathway Analysis software for core pathway analysis and DAVID to determine putative biological processes.

### mRNA expression analysis

Total RNA was extracted using Trizol reagent (Life Technologies) according to manufacturer’s instructions. 1 µg RNA was used to make cDNA using the qScript cDNA synthesis kit (Quanta Biosciences) according to manufacturer instructions. Relative levels of cDNA was measured by quantitative real time PCR using SYBR green Taq polymerase (Life Technologies) for 40 cycles of 95°C for 15 s, 60°C for 1 min using the StepOnePlus real-time PCR machine.

### Western blot analysis

Cells were lysed in lysis buffer [(50 mM Tris/HCl pH 7.5, 150 mM NaCl, 1 mM EDTA, 1 mM EGTA, 1% Triton X-100, and 10% glycerol containing phosphatase inhibitor (Thermo Scientific) and a mixture of protease inhibitors (Roche)]. Protein lysates (30 µg) were loaded and separated on 4-12% Bis Tris Novax NuPAGE gradient gels (Life Technologies), transferred to PVDF membrane, and incubated in blocking buffer [phosphate-buffered saline (PBS), 0.1% Tween 20 and 5% nonfat dried milk] for 1 h. To detect proteins of interest, membranes were incubated overnight at 4°C with primary antibodies. Immunoreactive bands were detected by incubating with horseradish peroxidase-conjugated secondary antibodies, followed by application of chemiluminescence substrate (Pierce ECL, Thermo Fisher Scientific). Membranes were exposed to either X-ray film (RPI) or Amersham Hyperfilm ECL (GE Healthcare) for signal detection before film development. To detect multiple proteins using the same membrane, membranes were stripped and re-probed with the appropriate primary antibodies. Quantification of the intensity from individual bands was done using Photoshop (Adobe). Normalized phospho-AKT (pAKT) signal was obtained by taking the ratio of pAKT and total AKT signals. AKT, pAKT-Thr308 (Cell Signaling Technologies), and Actin (Santa Cruz Biotechnology, Inc.) antibodies were diluted to working concentrations as described by manufacturers.

### Apoptosis/cell viability assay

To identify apoptotic cells, the Alexa Fluor 488 Annexin V/Dead Cell Apoptosis kit was used (Life Technologies). iMOP cells were treated with 25 µM LY294002 in the presence or absence of bFGF for 3 days before incubation with Alexa Fluor 488 Annexin V and propidium iodide (PI) according to manufacturer instructions. Cells labeled with Alexa Fluor 488 Annexin V and/or PI and were quantified by flow cytometry using a Beckman Coulter Gallios flow cytometer with the appropriate filters.

### Animals

B6;129S4 mice were used to produce P3-P6 pups for cochlear explants for untreated and small molecular treated samples. The PTEN conditional knockout (Backman et al., 2001), neural subset (NS) Cre (Ljungberg et al., 2009) and tdTomato mouse (Madisen et al., 2010) alleles have been previously described. P3-P6 pups were used to generate cochlear explants for untreated and genetamicin treated samples. Mice were kept in the Nelson Labs Animal Facility and used in accordance with the animal protocols.

### Cochlear explant cultures

Cochleae from P3-P6 pups were dissected and cleaned of surrounding tissue and bone. The stria vascularis was trimmed and the Reissner's and the tectorial membrane peeled away to expose the sensory epithelia. Since development of hair cells in the apex and base of the cochlea show significant variation in hair cell numbers, these portions of the cochlea were trimmed from the post-natal cochleae before *ex vivo* culture. After the apex and the base were trimmed, the entire cochlea was adhered onto a 1.5 cover glass treated with 10 µg/ml poly-L-ornithine and cultured in DMEM/F12 containing 10% FBS, 2 mM L-Glutamine, and 25 µg/ml carbenecillin. One day after plating, cochleae were treated with small molecules and drugs as described. Hair cells counts per 200 µm were used to determine the percent of hair cells.

### Immunofluorescence staining

Antibodies, dilutions and conditions used for immunostaining of iMOP cells were previously described (Kwan et al., 2015). MYO6 (Proteus Biosciences Inc.), GFAP (Dako) and TUBB3 (BioLegend) antibodies were purchased from commercial sources. Cochlear explants were fixed in 4% formaldehyde with 1× PBS for 1 h, permeabilized in wash buffer (PBS and 0.1% Triton X-100) for 10 min, incubated in blocking buffer (PBS, 10% goat serum and 0.1% Triton X-100) for 1 h and incubated overnight with a 1:500 dilution of MYO7A (Proteus Biosciences Inc.) primary antibody in blocking buffer. Cochleae were rinsed in wash buffer before incubating with a 1:500 dilution of goat anti-rabbit Alexa Fluor 488 secondary antibody and 1:500 phalloidin Alexa Fluor 647 (Life Technologies) in blocking buffer for 1 h. Cochleae were washed and mounted on slides with prolong gold antifade mounting media (Life Technologies). Immunofluorescence images were obtained using either a Zeiss 510 confocal microscope with a 40×1.3 NA water immersion objective or an Olympus DSU unit with a 60×1.3 NA apochromatic oil immersion objective. Conventional fluorescent filter sets were used. The relative percent of MYO7A positive inner hair cells (IHC) and outer hair cells (OHC) were obtained by calculating the ratio of MYO7A cells counted along the cochlear axis from experimental and control animals.

### Statistical analysis

All data are expressed as mean±standard deviation (s.d.) of values obtained from independent experiments. The numbers (*n*) of independent experiments are listed. Experiments using iMOP cells were performed with three technical replicates. For cochlear explant experiments, cochleae obtained from the same animal were considered technical replicates. In the cochlear explant experiments, each experiment was performed with two technical replicates. An unpaired two-tailed Student's *t*-test was used to determine statistical significance and associated with the appropriate *P* value. For all figures *P*-values are defined as: *P<0.05, **P<1×10^−2^, ***P<1×10^−3^ and ****P<1×10^−4^ unless otherwise stated.
